# Engineered *Chlamydomonas reinhardtii* Strains for Enhanced Astaxanthin Production

**DOI:** 10.3390/life15050813

**Published:** 2025-05-20

**Authors:** Federico Perozeni, Margherita Angelini, Matteo Ballottari, Stefano Cazzaniga

**Affiliations:** Department of Biotechnology, University of Verona, 37134 Verona, Italy; margherita.angelini@univr.it (M.A.); matteo.ballottari@univr.it (M.B.); stefano.cazzaniga@univr.it (S.C.)

**Keywords:** astaxanthin, microalgae, *Chlamydomonas reinhardtii*, metabolic engineering, carotenoids

## Abstract

Microalgae have evolved a diverse carotenoid profile, enabling efficient light harvesting and photoprotection. Previous studies have demonstrated the feasibility of genome editing in the green algal model species *Chlamydomonas reinhardtii*, leading to significant modifications in carotenoid accumulation. By overexpressing a fully redesigned β-carotene ketolase (bkt), the metabolic pathway of *C. reinhardtii* was successfully redirected toward astaxanthin biosynthesis, a high-value ketocarotenoid with exceptional antioxidant properties, naturally found in only a few microalgal species. In this study, a tailor-made double knockout targeting lycopene ε-cyclase (LCYE) and zeaxanthin epoxidase (ZEP) was introduced as a background for bkt expression to ensure higher substrate availability for bkt enzyme. The increased zeaxanthin availability resulted in a 2-fold increase in ketocarotenoid accumulation compared to the previously engineered bkt1 or bkt5 strain in the UVM4 background. Specifically, the best Δzl-*bkt*-expressing lines reached 2.84 mg/L under low light and 2.58 mg/L under high light, compared to 1.74 mg/L and 1.26 mg/L, respectively, in UVM4-*bkt* strains. These findings highlight the potential of rationally designed microalgal host strains, developed through genome editing, for biotechnological applications and high-value compound production.

## 1. Introduction

Carotenoids are liposoluble pigments synthesized by plants and microorganisms [1], playing crucial roles in photosynthetic organisms, including light harvesting and photoprotection [2]. These 40 carbon molecules are tetraterpenes composed of eight isoprene units, absorb light in the 350–550 nm range, and are characterized by a typical yellow, orange, or red coloration [3]. Carotenoids can be classified into two main groups: carotenes, which are hydrocarbons, and xanthophylls, which are their oxygenated derivatives. Among carotenoids, astaxanthin (3,3′-dihydroxy-β,β-carotene-4,4′-dione) is a ketocarotenoid with a reddish-purple hue and exhibits exceptionally high activity against ROS. Astaxanthin is synthesized from zeaxanthin through a reaction catalyzed by the enzyme beta-carotene ketolase (BKT) or from β-carotene via a dual reaction catalyzed by BKT and beta-carotene hydroxylase (CHYb), with canthaxanthin as an intermediate [4].

Astaxanthin is considered one of the most potent natural antioxidants, estimated to be approximately 6000 times stronger than vitamin C [5]. Oxidative stress is a major factor in most neurodegenerative diseases, including Alzheimer’s, Parkinson’s, and amyotrophic lateral sclerosis [6]. For that reason, astaxanthin has multiple health benefits due to its activity against oxidative stress [7]. Astaxanthin has been explored for its potential as an antitumor agent and for its role in preventing cardiovascular and neurological diseases, as well as diabetes [7]. It is also considered beneficial for patients at risk of ischemia, hypertension, and stroke, and it has shown improvements in memory in cases of vascular dementia [8].

Astaxanthin is produced only by a limited number of microalgal species. At the industrial scale, the demand for natural astaxanthin is currently met primarily by *Haematococcus lacustris* (formerly *H. pluvialis*), a green alga capable of accumulating up to 5% of its dry weight in ketocarotenoids under stress conditions [9]. Besides the massive astaxanthin accumulation, stress conditions also result in the degradation of photosynthetic machinery [10] and the formation of cysts surrounded by a thick cell wall [11]. Despite its high astaxanthin accumulation, using *H. lacustris* presents several challenges. Unlike other industrially cultivated algae, it requires a strictly controlled two-stage cultivation process, which increases production costs [12]. Its thick cell wall also necessitates energy-intensive extraction methods to enhance bioavailability for food and feed applications [13]. Astaxanthin can also be synthesized from petrochemical-derived substrates. While this method is economically advantageous, it has notable drawbacks, including producing a racemic mixture with significantly lower antioxidant capacity. Furthermore, due to its synthetic origin and the chemical processes involved, synthetic astaxanthin has not been approved by the FDA for direct use as a dietary supplement, and it can be used only as feed additive [14].

To overcome these limitations, genetic engineering strategies have been developed to enable astaxanthin production in diverse biotechnological host organisms, including bacteria [15,16] and yeast [17,18,19]. Due to their ability to exploit solar energy, photosynthetic organisms were also considered, including plants [20,21,22], cyanobacteria [23,24], and microalgae [25,26,27,28,29,30,31,32,33]. The microalga *C. reinhardtii* was deeply engineered to produce astaxanthin; the synthetic redesign of the endogenous pseudogene β-carotene ketolase (bkt) resulted in high-level ketocarotenoids accumulation [27]. More recently, in the same host, the overexpression of bkt along with two other limiting enzymes, β-carotene hydroxylase (CHYB) and phytoene synthase (PSY), resulted in the production of 23.5 mg L^−1^ (1.09 mg L^−1^ h^−1^), representing a fourfold increase compared to previous reports [25,34]. In *C. reinhardtii*, different xanthophylls are present: the β–β xanthophylls zeaxanthin, antheraxanthin, violaxanthin, and neoxanthin, and the ε–β xanthophylls loroxanthin and lutein [35]. All the xanthophylls are derived from β-carotene: lycopene β-cyclase (LCYB) converts lycopene into β-carotene, the first xanthophyll in the β-branch, while lycopene ε-cyclase (LCYE) utilizes the same substrate to generate γ-carotene, initiating the α-branch [36,37]. Under normal light, zeaxanthin does not steadily accumulate but is immediately converted into violaxanthin by the enzyme zeaxanthin epoxidase (ZEP) through transient mono-epoxidated antheraxanthin [37]. When the light intensity increases, a portion of violaxanthin is converted back into zeaxanthin in *C. reinhardtii* by the enzyme CVDE [38], by other VDE or VDE-like enzymes in other organisms [39]. The availability of substrates, such as zeaxanthin and β-carotene, is essential for astaxanthin synthesis. Tailor-made background strain with different carotenoid compositions can be helpful for specific metabolic engineering approaches to improve astaxanthin accumulation. For instance, Kneip and colleagues applied CRISPR-Cas genome editing to generate a background with increased accumulation of BKT substrates by knocking out the LCYE, leading to a 2.3-fold increase in astaxanthin accumulation compared to UVM4, which is widely used for its positive effect on gene expression due to the absence of the histone deacetylase Sir2 [40]. These findings underscore the potential of genome editing to develop tailored backgrounds that enhance astaxanthin production. Here, we report the use of another mutant, generated by the deletion of both LCYE and zeaxanthin epoxidase (ZEP) [41]. The deletion of these two enzymes significantly impacts the photosynthetic apparatus, resulting in decreased growth rate under low light conditions but increased productivity upon exposure to high light. This mutant strain is particularly beneficial due to its ability to accumulate high levels of both zeaxanthin and β-carotene, which are essential for efficient BKT-mediated astaxanthin synthesis. This tailored background ensures a higher availability of BKT substrates, thereby optimizing astaxanthin production. Moreover, certain sectors, such as aquafeed, require pure keto-carotenoids without contamination from other carotenoids, as the presence of other pigments may lead to unwanted coloration in the animal or interfere with product quality.

To assess the exclusive effect of the host strain, the chosen approach was to overexpress only the BKT enzyme, considering the existing shot of carotenoid biosynthesis toward zeaxanthin production. The selected background supported astaxanthin synthesis by providing a higher amount of BKT substrates compared to non-mutated strains, making it a promising candidate for genetic engineering.

## 2. Materials and Methods

### 2.1. Algal Cultivation and Strain Maintenance

The *C. reinhardtii* Δzl strain and its background cc4349 was kindly provided by Prof. EonSeon Jin [41]. All strains were maintained on Tris-Acetate-Phosphate (TAP) using ammonia as nitrogen source [42] agar plates or in liquid shake flasks at 25 °C, under continuous white light at an intensity of 80–100 µmol photons/m^2^/s. Growth tests were performed in shaking flasks at 25 °C, with cultures exposed to either low-light (70 µmol photons/m^2^/s) or high-light (750 µmol photons/m^2^/s) conditions. Microalgae were grown in TAP medium for mixotrophic conditions [43].

### 2.2. Expression Cassette, Transformation and Screening

The cloning vector used in this study contains the same genetic elements described in [27]. Specifically, the *C. reinhardtii* bkt coding sequence was modified by removing the last 345 bp, followed by synthetic redesign with codon optimization and intron spreading. The optimized sequence was then cloned into pOpt2_PsaD_mVenus_Paro, allowing the bkt protein to be fused with the photosystem I reaction center subunit II (PsaD) chloroplast targeting peptide and a C-terminal mVenus (YFP) tag. This assembled bkt fusion protein was previously shown to be correctly localized within the chloroplast, where it successfully catalyzed the biosynthesis of astaxanthin and canthaxanthin [27]. Nuclear transformation was carried out by glass bead methods [44]. A selection of transformants was performed on TAP agar plates supplied by paromomycin (12 mg/L) for 5–7 days. The initial screening of expressing colonies was performed by visually selecting 62 colonies displaying orange/brown pigmentation. These selected lines then underwent two rounds of 80% acetone extraction, followed by absorption spectra analysis and spectral fitting as described in the following sections.

### 2.3. Pigment Analyses

Pigments were extracted from intact cells using 80% acetone and analyzed through absorption spectroscopy and reverse-phase HPLC. Absorption spectra were recorded using a Jasco V-550 UV/VIS spectrophotometer (Jasco, Easton, MD, USA) and fitted following the method described by Croce et al. (2002) [45], with the inclusion of the astaxanthin absorption [27]. Given the similar absorption spectra of astaxanthin and canthaxanthin, the fitting was considered representative of the total ketocarotenoid content. Cell densities to estimate cellular ketocarotenoid contents were measured using a Countess II FL Automated Cell Counter (Thermo Fisher Scientific, Waltham, MA, USA). Reverse-phase HPLC was performed following the method described by Lagarde et al. (2000) [46]. The system was equipped with a C18 column and operated with a 15 min gradient of ethyl acetate (0–100%) in a 9:1:0.01 (vol/vol/vol) mixture of acetonitrile, water, and triethylamine, at a flow rate of 1.5 mL/min. Ketocarotenoid peaks were identified by comparing retention times and spectral profiles with commercial standards from CaroteNature GmbH (Münsingen, Switzerland). Specifically, spectral fitting was used during the screening phase to identify the highest accumulating strains, while HPLC was employed to precisely determine the pigment distribution on random colonies.

### 2.4. Growth Analysis

Microalgal growth was monitored following 720 nm Optical Density (720 nm OD) and cell dry mass. Optical density was measured using a Jasco V-550 UV/VIS spectrophotometer while dry biomass was evaluated by overnight lyophilization and gravimetric determination [27].

## 3. Results

### 3.1. Overexpression of bkt in Δzl Strains Resulted in Astaxanthin Accumulation

**Figure 1:**
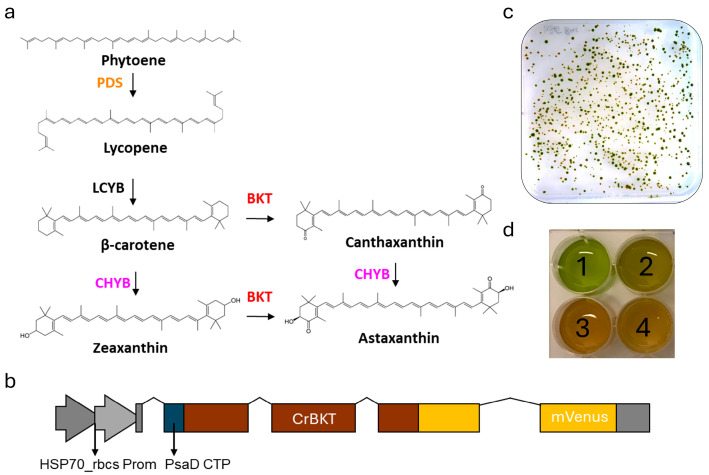
(**a**) Schematic of the carotenoid pathway towards astaxanthin biosynthesis in Δzl background. Only major carotenoids and steps are indicated. Name of enzymes are reported. BKT, carotene β-ketolase; CHYB, carotene β-hydroxylase; LCYB, lycopene β-cyclase; PDS, phytoene desaturase. (**b**) Schematic of the expression cassette used. CrBKT as previously was deprived of last 115aa, codon optimized, intron inserted and targeted into chloroplast with PsaD transit Peptide; mVenus (YFP) was used as fluorescent phusion protein and the gene expression was driven by the hybrid HSP70A-Rbcs2 promoter. (**c**) Picture of orange/brown colonies phenotype. (**d**) Liquid cultivation of (1) Δzl, (2) UVM4-bkt (bkt1) (3, 4) Δzl-bkt.

**Figure 2:**
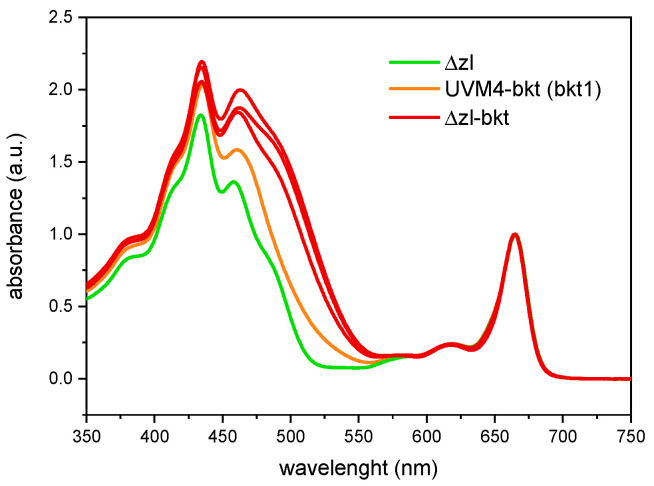
Absorption spectra of 80% acetone-extracted pigments from Δzl (green), three random Δzl-bkt transformed lines (red) and UVM4-bkt (bkt1, orange). Spectra are normalized to absorption in Qy region.

**Figure 3:**
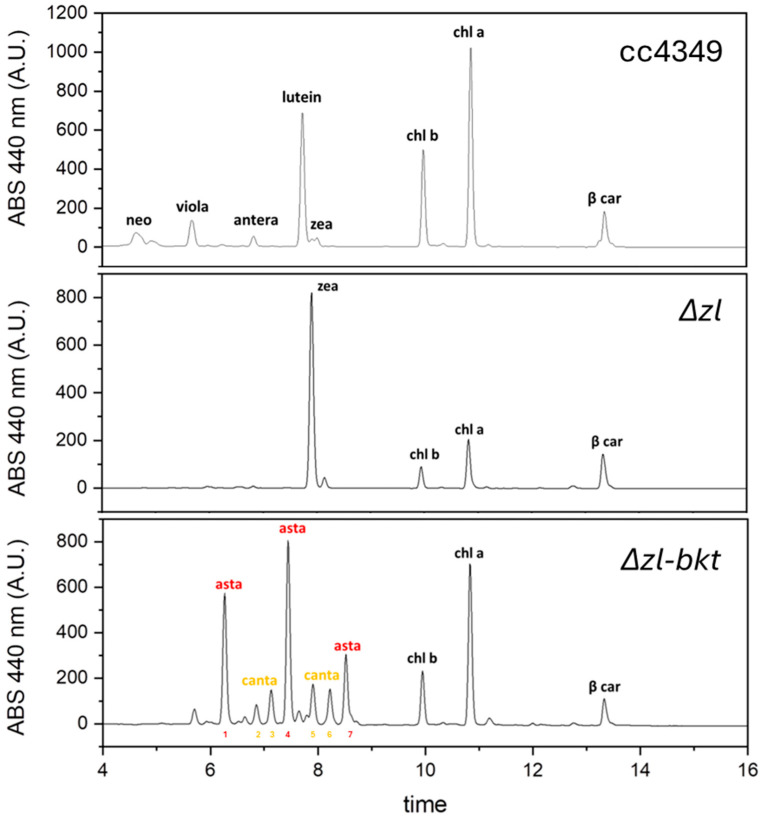
Representative HPLC of pigments from cc4349, Δzl and Δzl-bkt strains. Chromatograms are shown reported the absorbance (ABS) of the different peaks at 440 nm. Neo: neoxanthin, viola: violaxanthin, antera: antheraxanthin, zea: zeaxanthin, chl b: chlorophyll b, chl a: chlorophyll a; β-car: β-carotene. Astaxanthin (Asta, peaks: 1, 4 and 7) and canthaxanthin (cantha, peaks: 2, 3, 5 and 6) peaks are individually labeled.

**Figure 4:**
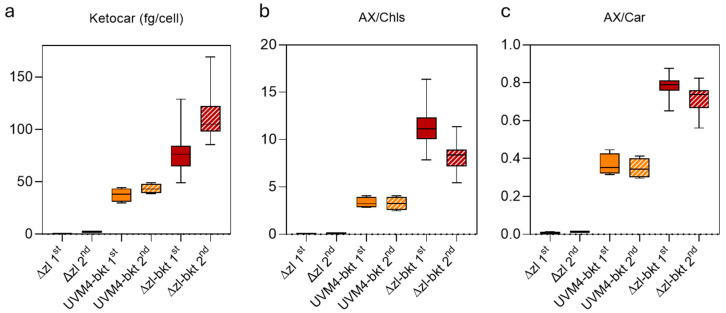
First and second screenings were performed by fitting absorption spectra to quantify ketocarotenoid content per cell (**a**), as well as in relation to chlorophylls (**b**) and carotenoids (**c**). Δzl (*n* = 4), UVM4-bkt (*n* = 8), and Δzl-bkt (*n* = 62 in first screening and *n* = 20 in second screening) were analyzed.

The Δzl mutant was obtained through CRISPR-Cas-mediated double knockout of the LCYE and ZEP genes [41]. As a result of these mutations, the strain exclusively accumulates zeaxanthin as its sole xanthophyll (Figure 1a) since the inactivation of *LCYE* blocks the α-branch of carotenoid biosynthesis, while the knockout of *ZEP* prevents the conversion of zeaxanthin to violaxanthin, effectively channeling the carotenoid flux toward zeaxanthin accumulation. Characterization of the Δzl mutant revealed an adaptive advantage under high-light photoautotrophic conditions, attributed to the zeaxanthin scavenging activity [47]. Given the increased availability of zeaxanthin, directly accessible to the bkt enzyme, and the absence of growth impairments, the Δzl mutant was selected as the optimal background for astaxanthin production. Additionally, the feasibility of using a fully redesigned enzyme in *C. reinhardtii* has been previously demonstrated [27]. In this study, we employed the same expression cassette. Specifically, as illustrated in Figure 1b, the bkt coding sequence was codon-optimized, and rbcs intron 1 was strategically inserted along the gene to enhance expression [48]. Moreover, the bkt sequence was fused to mVenus, generating a fusion protein, as previously described [27]. The assembled vector was introduced into the Δzl mutant genome via the glass bead transformation method, as detailed in the Materials and Methods section. Transformed cells were selected on TAP medium supplemented with 12 µg/mL paromomycin, and colonies emerged after six days. Notably, some colonies exhibited a distinct orange-brown pigmentation (Figure 1c). The same altered pigmentation was observed when individual colonies were cultivated in liquid medium (Figure 1d). To identify the BKT expressing lines with the highest ketocarotenoid accumulation, we initially selected 62 colonies based on the intensity of their red coloration. These colonies then underwent acetone extraction, followed by carotenoids quantification by spectral analysis. The pigment absorption spectra from Δzl-bkt lines, extracted with 80% acetone, displayed a characteristic shoulder above 500 nm, which was absent in the parental Δzl strain (Figure 2, showing three representative colonies out of the 62 analyzed). This spectral feature corresponds to the absorption peak of ketocarotenoids, such as astaxanthin. After normalization in the Qy region, Δzl-bkt showed a more pronounced shoulder compared to the previously generated bkt1 line in the UVM4 background [27] suggesting either a higher accumulation of ketocarotenoid or a reduction in chlorophyll content. HPLC analysis of Δzl-bkt, Δzl and the WT strains (cc4349) are reported in Figure 3. As expected, zeaxanthin was the only xanthophyll present in the Δzl. Differently, the Δzl-bkt chromatogram exhibited additional peaks not present in WT or in Δzl which can be attributed to astaxanthin and canthaxanthin with astaxanthin being 75.8% of total ketocarotenoid, a value comparable with previous finding when bkt gene was overexpressed in UVM4 background [27]. Ketocarotenoid (keto) quantification in Δzl-bkt lines and the two highest-accumulating UVM4-bkt lines (bkt1 and bkt5) was performed through absorption spectra fitting as previously reported [27]. Specifically, keto accumulation was assessed in relation to cell density (keto/cells), chlorophyll content (keto/Chls), and total carotenoid content (keto/Car) across all 62 screened Δzl-bkt lines, as well as in Δzl and UVM4-bkt strains. As shown in Figure 4a–c, the Δzl strain, which lacks the bkt enzyme, did not accumulate ketocarotenoids. Conversely, both UVM4-bkt and Δzl-bkt strains exhibited keto accumulation. Notably, accumulation per cell was nearly twice as high in Δzl-bkt compared to UVM4-bkt (76 fg/cell vs. 37 fg/cell, respectively). Further analysis of ketocarotenoids accumulation relative to chlorophylls and carotenoids confirmed that Δzl-bkt lines accumulated twice as much keto as UVM4-bkt, which represent 78% of total carotenoids. However, it is important to note that keto/Chl and keto/Car ratios can be influenced by variations in chlorophyll or total carotenoid content. Previous studies have shown that bkt expression in *C. reinhardtii* leads to a significant reduction in chlorophyll content [27]. Therefore, using keto/Chl or keto/Car ratios could result in variations that are not directly attributable to changes in ketocarotenoids themselves, but rather to differences in chlorophyll or carotenoid levels. For this reason, keto/cells was prioritized as the primary screening parameter. This parameter provides an absolute quantification of astaxanthin per cell, which is directly related to the volumetric productivity of the strain, reflecting the overall growth and biomass accumulation. Screening of the 62 Δzl-bkt lines revealed significant variability in astaxanthin content per cell, ranging from 50 to 121 fg/cell. This variability is attributed to the insertional position effect, as the bkt expression cassette was randomly integrated into the *C. reinhardtii* nuclear genome, and the insertion site can either enhance or suppress gene expression. Following this initial screening, the 20 lines with the highest keto/cell accumulation were selected and further cultured in TAP medium for additional evaluation. The same parameters, keto/cell, keto/Chls, and keto/Car, were evaluated for the 20 selected Δzl-bkt lines (Figure 4a–c). Considering only these top-20 selected Δzl-bkt lines, the average ketocarotenoids content per cell increased from 76 fg/cell to 115 fg/cell, representing a 164.6% increase compared to UVM4-bkt lines.

It is important noting that the UVM4-bkt lines herein analyzed, bkt1 and bkt5, were selected as the top-performing lines in the UVM4 background. As observed in the first screening phase, Δzl-bkt lines accumulated ~2.5 times more ketocarotenoids per cell than UVM4-bkt (118 fg/cell vs. 43 fg/cell). However, the trends for keto/Chls and keto/Car differed between screenings. While keto/cell increased, both keto/Chls and keto/Car ratios decreased, with keto/Chls dropping by 27% and keto/Car decreasing by 9% in the top-20 Δzl-bkt lines compared to the values obtained considering the initial screening on all the 62 expressing lines. This reduction in keto/Chls and keto/Car is attributed to the screening criteria: keto/cells was prioritized to minimize variations caused by differences in chlorophyll and total carotenoid content. The fact that the top 20 selected lines did not rank highest also for keto/Chls or keto/Car further validates the effectiveness of keto/cell as the primary screening parameter. Among the 20 selected Δzl-bkt lines, two (M37, M62) were identified as the best performers in terms of keto/cell content and were chosen for further analysis.

### 3.2. Algal Growth Is Not Perturbed by Ketocarotenoids Presence

**Figure 5:**
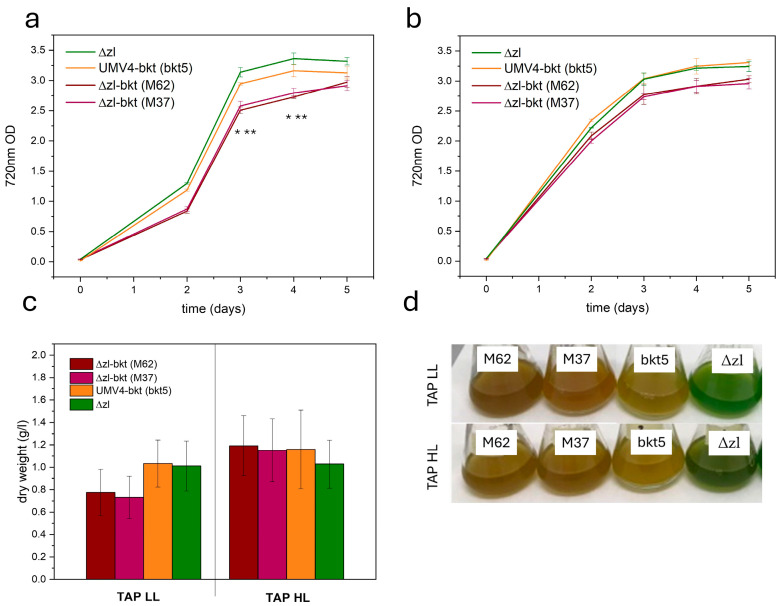
Growth curves of Δzl-bkt (M62-M37), UVM4-bkt (bkt1), and Δzl in TAP medium using low (70 µmol/m^2^/s) (**a**) or high (750 µmol/m^2^/s) (**b**) light. Curves were obtained by measuring the optical density (O.D.) at 720 nm. Error bars are reported as standard deviation (*n* = 3). Values significantly different compared to UVM4-bkt are reported with * in the case of M62 and ** for M37 (Student’s *t* test, *p* < 0.05). (**c**) Dry weight obtained at the end of the growth curve. Error bars are reported as standard deviation (*n* = 3). (**d**) Orange/red phenotype of microalgae growth in both conditions.

The impact of ketocarotenoid accumulation in *C. reinhardtii* Δzl-bkt was assessed by cultivating selected transformant lines (M62 and M37), along with Δzl and UVM4-bkt (bkt5), in 50 mL flasks under mixotrophic conditions using TAP medium [43] at either 70 or 750 µmol/m^2^/s, as described in Materials and Methods. Under low light conditions, both Δzl-bkt expressing lines exhibited reduced growth compared to Δzl and bkt1 (Figure 5a,c). However, this difference was not observed under high light intensity (Figure 5b,c), where both 720 nm OD and dry weight showed no difference. The altered pigmentation noted during screening was also evident, with Δzl-bkt lines displaying a more pronounced orange coloration than bkt5 in both conditions (Figure 5d). Given the previously reported reduction in chlorophyll content in UVM4-bkt, photosynthetic pigment content was analyzed under both conditions to investigate the potential cause of Δzl-bkt impaired growth. Table 1 presents the pigment content of Δzl-bkt M62, UVM4-bkt, and Δzl. Growth in TAP under low-light conditions led to a significant reduction in cellular chlorophyll content compared to Δzl background. A similar decrease was previously observed in the bkt1 line, which exhibited ~3.5-fold lower chlorophyll levels than its UVM4 background [27]. However, in Δzl-bkt, the decrease in chlorophyll content was even more pronounced, with chlorophyll content decreasing by an additional 40% compared to bkt1. Despite this drastic decline, the Chl/Car ratio was only slightly affected, remaining comparable to bkt1 but lower than in Δzl. In addition, Δzl-bkt lines displayed a markedly higher Chl a/b ratio than both bkt1 and Δzl, suggesting a reduction in photosystem antenna complexes, where Chl b is primarily associated with peripheral light-harvesting proteins. When cells were grown under high-light conditions, Δzl-bkt and bkt5 lines showed comparable chlorophyll content, with both exhibiting an increased Chl a/b ratio; although, the increase was less pronounced than under low light. In this condition, however, the Chl/Car ratio was lower in Δzl-bkt lines compared to bkt5.

### 3.3. Yield of Ketocarotenoids in Different Growth Conditions

**Figure 6:**
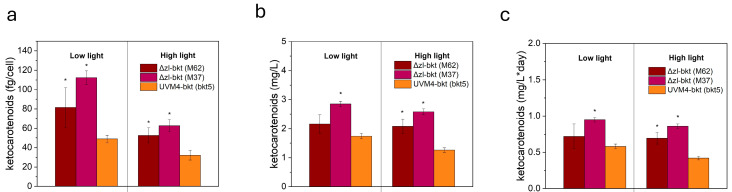
Quantification of ketocarotenoids per cell (**a**), volumetric productivity (**b**), and daily productivity (**c**). Data were obtained from spectral fitting of 80% acetone extracts. Error bars are reported as standard deviation (*n* =3). Values significantly different compared to UVM4-bkt are reported with * (Student’s *t* test, *p* < 0.05).

The accumulation of ketocarotenoids was assessed in cells grown in low-light or high-light conditions. As shown in Figure 6a, both M62 and M37 exhibited higher ketocarotenoid levels per cell compared to bkt5, with a more pronounced increase in M37 under low-light conditions. Specifically, in this condition, M37 accumulated more than twice the amount of keto/cell, reaching an average of 112 fg/cell, compared to 48.9 fg/cell in bkt5. In high-light conditions, all strains displayed an overall ~50% reduction in ketocarotenoid content per cell. However, M37 consistently showed the highest keto/cell content accumulation, maintaining the same relative difference observed under low-light conditions. Both M62 and M37 exhibited impaired growth under low light in TAP, resulting in reduced OD at 720 nm and lower biomass accumulation. Consequently, despite the significantly higher ketocarotenoid accumulation per cell in low light compared to high light, the overall volumetric productivity remained similar, reaching 2.84 mg/L in low light and 2.58 mg/L in high light for the best-performing line, M37 (Figure 6b). In contrast, the growth of bkt5 was comparable in both conditions, leading to similar volumetric ketocarotenoid production (1.74 mg/L in low light and 1.26 mg/L in high light) (Figure 6b). Nevertheless, despite the decreased growth observed in low light for M62 and M37, their volumetric productivity remained higher than that of bkt5 in both conditions. On average, ketocarotenoid titer in M37 was 2.84 mg/L in low light and 2.58 mg/L in high light, compared to 1.74 mg/L and 1.26 mg/L, respectively, in bkt5, corresponding to an increase of approximately 63% in low light and 104% in high light. Lastly, since all strains reached the stationary phase within the same timeframe under both conditions, this trend was also reflected in daily productivity, with M37 achieving the highest value in low light at 0.94 mg ketocarotenoids/L/day (Figure 6c).

The proportion of total carotenoids converted into ketocarotenoids was calculated and is reported in Table 2. Both M62 and M37 exhibited high conversion efficiency, with 60% to 71% of total carotenoids transformed into ketocarotenoids. In contrast, bkt5 achieved only 29% conversion in low light and 40% in high light.

These results demonstrate that a tailor-made genetic background can effectively enhance the biosynthesis of high-value ketocarotenoids in *C. reinhardtii*. The increased accumulation is likely due to greater substrate availability, representing a significant step toward optimizing Chlamydomonas as a robust cell factory.

## 4. Discussion

Currently, natural astaxanthin is primarily produced by *Haematococcus lacustris*, a green microalga capable of accumulating large amounts of the compound under stress conditions. However, its industrial use faces several challenges, including slow growth rates, complex cultivation requirements, and costly downstream processing, which limit its scalability and economic viability. Synthetic astaxanthin is less expensive but has lower bioactivity and is not approved for direct human consumption, limiting its use as a feed additive to provide pigmentation. Genetic engineering has been explored in various organisms, including bacteria, yeast, plants, cyanobacteria, and microalgae, to create alternative platforms for astaxanthin production.

In this study, we investigate the use of a tailor-made background created by CRISPR-Cas, introducing a double knockout in the lycopene ε-cyclase (LCYE) and zeaxanthin epoxidase (ZEP) genes, referred to as the Δzl mutant. This double mutation forces the accumulation of only zeaxanthin and β-carotene, which serve as substrates for ketocarotenoid biosynthesis. A similar approach was previously employed using a single knockout in LCYE, thus increasing the availability of zeaxanthin and β-carotene by removing the competitive pathway leading to lutein and loroxanthin biosynthesis. In this background, by overexpressing three key enzymes in the astaxanthin biosynthesis pathway, BKT, CHYb, and phytoene synthase (PSY), astaxanthin accumulation was achieved, obtaining a titer of 1.8 mg/L, about a 2.3-fold increase compared to the case of using UVM4 background [40].

In the present work, we used a simpler approach, overexpressing only the BKT enzyme in the Δzl background, as previously done in the case of the genetic engineering of the UVM4 background by the overexpression of the BKT enzyme [27]. The Δzl-bkt strain (M37) accumulated 2.84 mg/L of total ketocarotenoids under low light and 2.58 mg/L under high light, representing a 60% and 104% increase, respectively, compared to the UVM4-bkt strain. These results demonstrate a significant improvement in ketocarotenoid accumulation compared to the UVM4 background.

High-performance liquid chromatography (HPLC) analysis revealed that in Δzl-bkt lines all zeaxanthin was converted into astaxanthin, representing approximately 75% of the total ketocarotenoids. The remaining 25% was canthaxanthin, produced by BKT acting on β-carotene. This astaxanthin/canthaxanthin ratio mirrors that previously observed in UVM4 strains, highlighting the limited availability of CHYb in the astaxanthin biosynthesis pathway, being the conversion of canthaxanthin to astaxanthin catalyzed by the CHYb enzyme, and only in the case of overexpression, a higher Asta/Cantha ratio could be reached [25]. Furthermore, due to the simplified carotenoid pool available, the Δzl-bkt strains were able to convert a higher proportion of the total carotenoid pool into ketocarotenoids, with a conversion rate of about 70% in the best case, compared to a maximum of 40% in the bkt5 strain.

Interestingly, the Δzl-bkt lines showed relatively stable carotenoid conversion rates when shifting from low to high light, with an 8% increase in M62 and a 7% decrease in M37. In contrast, the bkt5 strain exhibited a 38% higher conversion of carotenoids into ketocarotenoids under high light. This difference can be explained by substrate availability: in the Δzl mutant, only zeaxanthin and β-carotene are present, whereas in the UVM4 strain, the full set of Chlamydomonas pigments is available, with zeaxanthin only appearing under high light due to the xanthophyll cycle [49].

Finally, the Δzl-bkt strains showed a non-significant but noticeable reduction in biomass accumulation in low light conditions compared to the bkt overexpressing line in the UVM4 background, indicating a potential growth disadvantage in limiting light conditions. This is consistent with previous findings that the Δzl mutant experiences growth challenges under photoautotrophic conditions in low light, but performs better under high light [47]. This phenomenon can likely be attributed to the reduced antenna size in the Δzl mutant, which decreases light harvesting efficiency. This further destabilization of the antenna system in Δzl-bkt strains is reflected in the chlorophyll a/b ratio, which is further increased in the overexpressing line. Differently, in high-light conditions, the growth kinetic of Δzl-bkt strains was similar to the bkt5 case.

In both low-light and high-light conditions, the Δzl-bkt strains achieved significantly higher daily ketocarotenoid production than the bkt5 strain (Figure 6c). The higher ketocarotenoid productivity and higher conversion rate of carotenoids into ketocarotenoids (Table 2) of Δzl-bkt strains make these strains an attractive candidate for industrial-scale ketocarotenoid extraction. However, for the successful transition from laboratory-scale to industrial-scale applications, scalability remains a critical consideration. This involves not only ensuring that the strains maintain high productivity under large-scale cultivation conditions. Further testing under high-stress conditions, such as intense high-light exposure (3000 µmol·m^−2^·s^−1^) or industrial scaling up, are needed to assess the strain’s survival and performance under these conditions. These tests will provide valuable insights into the robustness of the strains and their ability to thrive under the varied conditions typical of large-scale, cost-efficient production systems.

In conclusion, this study reinforces the potential of *C. reinhardtii* as a versatile and robust platform for the sustainable production of high-value bioactive molecules such as ketocarotenoids.

In recent years, *C. reinhardtii* has been successfully engineered to produce a growing array of commercially relevant compounds, including therapeutic peptides, recombinant proteins, and other high-value terpenoids [50,51].

By demonstrating stable and efficient production of ketocarotenoids in multiple genetic backgrounds, this study reinforces the positioning of *Chlamydomonas reinhardtii* as a promising biotechnological chassis for industrial applications. While *C. reinhardtii* strains capable of astaxanthin accumulation have already been developed, this work provides an important step forward by enhancing productivity in alternative genetic contexts, broadening the versatility and robustness of the platform. The strains we have generated show increased capacity for ketocarotenoid accumulation, further reducing production costs and positioning *C. reinhardtii* as an even more competitive option in the market for aquafeed. By offering a higher accumulation of ketocarotenoids, these strains can contribute to the production of feed products that not only ensure improved animal welfare but also support a shift away from synthetic chemical processes derived from petroleum-based sources.

For certain applications, such as animal feed or nutraceuticals, it is crucial to achieve high concentrations of the desired pigment, with minimal to no presence of undesired pigments that may cause unwanted coloration in the animal or interfere with product quality. The strains we have developed in *C. reinhardtii* are well-suited to meet this demand, providing high-purity ketocarotenoids due to the improvements made in the genetic background.

The growing role of *C. reinhardtii* in biotechnological applications reflects its ability to contribute to the circular bioeconomy, reducing reliance on fossil fuels and minimizing environmental impact. By producing valuable bioactive compounds in an environmentally friendly manner, *C. reinhardtii* opens new opportunities for more efficient bioproduction across various sectors, supporting the ongoing shift toward sustainable industrial practices and green biotechnologies. This work adds another piece to the puzzle of producing sustainable and high-quality animal feed, as well as nutraceuticals, in an eco-friendly, cost-efficient, and scalable manner, fully decoupled from the harmful environmental impacts of petroleum-based processes.

## Figures and Tables

**Table 1:** Pigment content of Δzl-bkt, UVM4-bkt, and Δzl. Pigment content determined in cells grown under low (LL, 70 µmol/m^2^/s) or high (HL, 750 µmol/m^2^/s) light in TAP medium. Data are expressed as mean ± SD (*n* = 4).

		Δzl-bkt (M62)			UVM4-bkt (bkt5)			Δzl		
TAP LL	pg Chl/cell	0.43	±	0.04	0.70	±	0.04	0.88	±	0.06
	Chla/Chlb	4.55	±	0.48	2.32	±	0.08	2.61	±	0.10
	Chl/Car	2.09	±	0.16	2.65	±	0.07	3.2	±	0.33
TAP HL	pg Chl/cell	0.19	±	0.01	0.24	±	0.05	0.54	±	0.08
	Chla/Chlb	3.72	±	0.09	2.55	±	0.33	2.83	±	0.19
	Chl/Car	1.24	±	0.01	1.93	±	0.01	2.18	±	0.09

**Table 2:** Carotenoid conversion into ketocarotenoid in TAP low light (LL, 70 µmol/m^2^/s) and high light (HL, 750 µmol/m^2^/s). Data were obtained by spectral fitting of 80% acetone extract. *n* = 3.

	Δzl-bkt (M62)			Δzl-bkt (M37)			UVM4-bkt (bkt5)		
TAP LL	60%	±	2%	71%	±	3%	29%	±	1.5%
TAP HL	65%	±	1.5%	66%	±	2.5%	40%	±	3%

## Data Availability

All data generated in this study are available in this published article.
